# Long-Range Electrostatic Colloidal Interactions and Specific Ion Effects in Deep Eutectic Solvents

**DOI:** 10.1021/jacs.1c04781

**Published:** 2021-08-30

**Authors:** Adrian Sanchez-Fernandez, Andrew J. Jackson, Sylvain F. Prévost, James J. Doutch, Karen J. Edler

**Affiliations:** † Food Technology, Engineering and Nutrition, Lund University, Box 124, 221 00 Lund, Sweden; ‡ European Spallation Source, Box 176, 221 00 Lund, Sweden; § Department of Physical Chemistry, Lund University, Lund, SE-221 00, Sweden; ∥ Institut Laue-Langevin, 71 Avenue des Martyrs, 38000, Grenoble, France; ⊥ ISIS Neutron and Muon Source, Science and Technology Facilities Council, Rutherford Appleton Laboratory, Didcot, OX11 0QX, U.K.; # Department of Chemistry, University of Bath, Claverton Down, Bath, BA2 7AY, U.K.

## Abstract

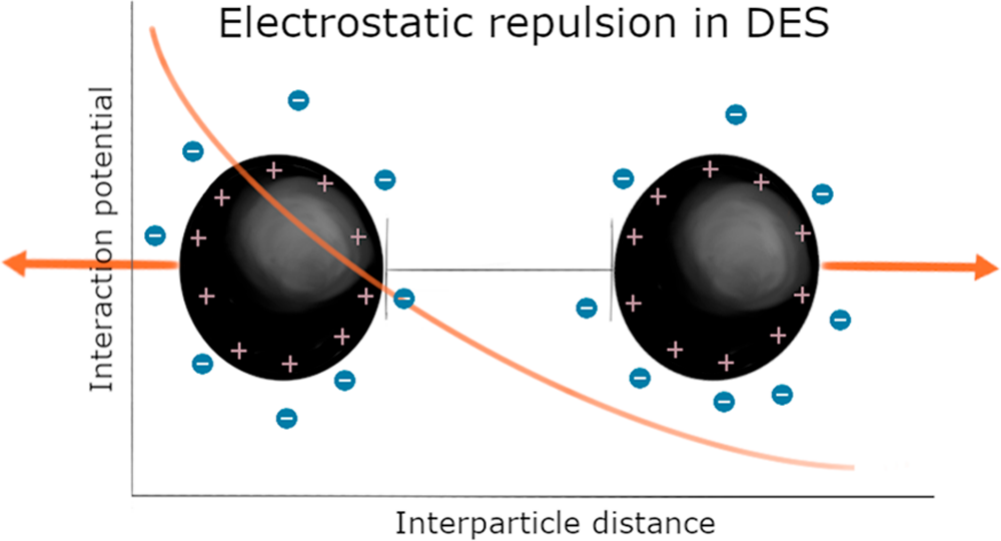

While the traditional consensus dictates that high ion concentrations lead to negligible long-range electrostatic interactions, we demonstrate that electrostatic correlations prevail in deep eutectic solvents where intrinsic ion concentrations often surpass 2.5 M. Here we present an investigation of intermicellar interactions in 1:2 choline chloride:glycerol and 1:2 choline bromide:glycerol using small-angle neutron scattering. Our results show that long-range electrostatic repulsions between charged colloidal particles occur in these solvents. Interestingly, micelle morphology and electrostatic interactions are modulated by specific counterion condensation at the micelle interface despite the exceedingly high concentration of the native halide from the solvent. This modulation follows the trends described by the Hofmeister series for specific ion effects. The results are rationalized in terms of predominant ion–ion correlations, which explain the reduction in the effective ionic strength of the continuum and the observed specific ion effects.

## Introduction

Electrostatic interactions play an essential role in biological and technological processes. From ion motion in batteries to protein function in living cells, charge modulation dictates the function of processes that involve ions.^1,2^ The classical approach to treat electrostatics considers ions as ideal point charges, where no ion–ion interactions occur as ions with no volume are considered to exist in a theoretically dilute regime.^3^ To account for the nonidealities arising from electrostatic forces between ions, Debye and Hückel formulated the theory that describes the interaction landscape in dilute electrolytes by adding an activity coefficient that quantifies such an interaction.^4^ Subsequently, DLVO theory was developed to quantitively describe the interplay between van der Waals attraction forces and electrostatic repulsion in colloidal systems.^5^ Although DLVO theory has been the benchmark framework to describe colloidal stability, it is well-known that electrostatics are driven not only by the ion charge but also by other complex mechanisms, e.g. the solvation free energy of the ions near macromolecules, specific charge–charge interactions, and Hofmeister effects.^6^ However, all these theories fail to accurately describe important biological and technological processes, as electrostatic interactions in concentrated ion solutions are far from the ideal behavior.^7^ Recent investigations have shown that ion–ion correlations at high ion concentrations, such as in concentrated electrolytes and ionic liquids, reduce the apparent ionic strength of the continuum.^8,9^ This prompts complex ionic systems to show a reversion in the Debye–Hückel theoretical predictions, and larger Debye lengths are observed with increasing ion concentrations at high ionic strength.

Deep eutectic solvents (DES) are sustainable liquids obtained and stabilized through the formation of an extensive hydrogen bond network often between an organic salt with a neutral molecule at a eutectic ratio specific to the mixture (e.g., 1 mol of choline chloride:2 mol of urea).^10−12^ The result from the complexation of the precursors is a solvent at room temperature. DES are promising sustainable alternatives to traditional molecular solvents in a variety of applications. For instance, these solvents have been proposed as environments for protein preservation, metal electrodeposition, and synthesis of nanostructured materials and as electrolytes in green lithium-ion batteries, among others.^13−17^ All of these applications share a common theme; i.e. electrostatics dictate the behavior of the systems. The theoretical ionic strength of DES is often higher than 2.5 M when all ions are assumed to be dissociated. Under these conditions, the predicted Debye length becomes no larger than the ion radius. Thus, long-range electrostatic interactions should be completely screened at these ion concentrations.^9^ However, DES may present another exception to the traditional framework for electrostatic interactions, as occurs with ionic liquids and other concentrated electrolytes. Thus, investigating the electrostatic interaction landscape in DES is of great relevance for understanding and developing better technologies based on these solvents and will also contribute to the development of knowledge about highly ionic systems.^8^

Small-angle scattering provides one of the very few methods that allows a direct probe of the correlations between colloidal particles (e.g., micelles). As such, modeling approaches have been developed to probe interaction potentials. Hard-sphere (HS) or Coulomb interaction potentials can be modeled from small-angle scattering data of concentrated samples, and the strength and decay profile of the interaction potential can be investigated.^18−22^ In this paper, we present an investigation of long-range interparticle interactions in DES. Also, the modulation of electrostatic forces due to the condensation of different counterions at the particle interface was probed. To probe colloidal interactions in DES, we measured the scattering from dodecyltrimethylammonium (C_12_TA^+^) micelles with different counterions (chloride, Cl^–^; bromide, Br^–^; nitrate, NO_3_^–^; and sulfate, SO_4_^2–^) at different volume fractions in 1:2 choline chloride:glycerol and 1:2 choline bromide:glycerol. The behavior of these surfactants has been extensively studied in aqueous solution and ionic liquids, thus providing a baseline comparison to the results presented here. Also, the results were compared to the theoretical predictions for hard spheres interacting through excluded volume effects.

## Experimental Section

### Materials

Protiated choline bromide (h-ChBr, TCI Chemicals, >98%), protiated glycerol (h-Glyc, Sigma-Aldrich, 99%), deuterated choline chloride (d-ChCl, CK Isotopes, 99%, 99.6% D), deuterated glycerol (d-Glyc, CK Isotopes, 99%, 99% D), protiated C_12_TAC (h-C_12_TAC, Sigma-Aldrich, >99%), protiated C_12_TAB (h-C_12_TAB, Sigma-Aldrich, 99%), and D_2_O (Sigma-Aldrich, 99.9% D) were purchased and used as received. Protiated 1:2 choline bromide:glycerol (h-ChBr:h-Glyc) and deuterated 1:2 choline chloride:glycerol (d-ChCl:d-Glyc) were prepared following the same protocol presented for the synthesis of protiated 1:2 choline chloride:glycerol.^12^ Solvents were freeze-dried, sealed, and stored under a dry atmosphere to minimize water absorption. Water content was determined using a Mettler-Toledo DL32 Karl Fischer titrator to an average content of 0.32 and 0.64 wt % for the chloride- and bromide-based solvents respectively, during the experimental procedure presented here.

Protiated C_12_TANO_3_ (h-C_12_TANO_3_) and C_12_TA(SO_4_)_1/2_ (h-C_12_TA(SO_4_)_1/2_) were prepared by exchanging the chloride counterion from C_12_TAC following the procedure presented in the Supporting Information (SI).^23^ Tail-deuterated dodecyltrimethylammonium bromide (d-C_12_TAB, d_25_) was prepared at the ISIS Deuteration Facility and used as received.

Samples for small-angle neutron scattering (SANS) measurements were prepared by dilution of stock solutions of the different surfactants in each solvent. Stock solutions were prepared by directly mixing each surfactant in the DES and equilibrated at 50 °C for 24 h. These stock solutions were diluted using pure DES to reach the desired final concentrations. The samples were equilibrated at 50 °C for 24 h, sealed, and stored under a dry atmosphere to prevent water adsorption. Samples in D_2_O were prepared using the same protocol without requiring the equilibration step.

### Methods

SANS experiments were performed on Sans2d (ISIS Pulsed Neutron Source, UK)^24^ and D11 (Institute Laue-Langevin, France).^25^ For both experiments, samples were loaded in 1 mm path length, 1 cm width, quartz Hellma cells. The cells were placed in a temperature-controlled sample changer at a constant temperature of 50 °C during measurement for the samples containing surfactants in DES. Surfactant samples in D_2_O were measured at 25 °C. Data reduction was performed using the standard protocols of each beamline accounting for sample transmission, detector efficiency, and the scattering from an empty cell, and resulting in data sets containing the absolute scattered intensities, *I*(*q*) in cm^–1^, versus momentum transfer, *q* in Å^–1^.^26,27^ The scattering of the solvents was subtracted as a background contribution accounting for the incoherent scattering from each sample. The theoretical models were smeared using a Gaussian function to account for instrument resolution.^28^

SANS data analysis was performed using a model-based approach. The small angle scattered intensity from an isotropic, centrosymmetric particle can be written as
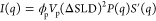
1where *ϕ*_p_ is the volume fraction of particles, *V*_p_ is the particle volume, and ΔSLD is the difference in the scattering length density (SLD) between particles and the solvent. The form factor, *P*(*q*), and the apparent interparticle structure factor, *S′*(*q*), are *q*-dependent functions that respectively describe the particle morphology and interparticle interactions. Previous investigations on the micellization of alkyltrimethylammonium bromide surfactants in choline chloride:glycerol DES have shown that the SANS data from micelles in the dilute regime can be satisfactorily modeled using a uniform ellipsoid form factor.^29,30^ As such, we have decided to use this model to account for the form factor of the micelles. The analytical model uses two structural parameters to describe the shape of the uniform ellipsoid, the equatorial radius perpendicular to the rotation axis of the spheroid, *r*_eq_, and the aspect ratio between the two radii of the spheroid, AR (AR = *r*_po_/*r*_eq_, where *r*_po_ is the polar radius parallel to the rotational axis of the spheroid). When AR > 1, this model describes an ellipsoid with a prolate distribution of mass.

The SLD of each component of the system was calculated by accounting for the neutron scattering length of the atomic group (*Σb*_*i*_) and the volume this occupies (*V*_m_). As the solvation of the headgroup and the chemical similarity between the quaternary ammonium headgroup and solvent components significantly reduces the scattering contribution of the headgroup region,^29^ the scattering signal is dominated by the micelle core for the isotopic mixtures used in these experiments. Therefore, the SLD of the micelle was calculated as equivalent to that of the lyophobic tail and fixed during fitting, neglecting possible solvation in the core by the DES. Molecular volumes, neutron scattering lengths, and SLDs used for the analysis of the data are presented in Table 1.

**Table 1:** Volumes, Neutron Scattering Lengths, and Scattering Length Densities of the Constituents of the Systems Studied Here

Unit	*V*_m_/Å^3^	∑b_i_/fm	SLD/×10^–6^ Å^–2^
d-ChCl:d-Glyc	434.9	281	6.40
h-ChBr:h-Glyc	434.9^a^	17.7	0.42
C_12_D_25_	350.2^b^	242	6.92
C_12_H_25_	350.2^b^	–13.7	–0.39
D_2_O	30.07	19.1	6.37

a The molecular volume of 1:2 choline bromide:glycerol has been approximated as the same as that of the 1:2 choline chloride:glycerol.^29^ This is a reasonable assumption based on the similar size of the anions (*r*_Cl_ = 181 pm; *r*_Br_ = 196 pm).^31^

b The molecular volume of the surfactant tail was calculated using the Tanford equations.^32^

The dynamic character of micellization is expected to follow a rapid association–dissociation equilibrium, as it does in aqueous solution. This results in a population of micelles with different aggregation numbers, which is predicted to follow a relatively narrow distribution for globular aggregates.^29,33^ A polydispersity function (*p*) was included to account for the nonuniformity of the micelles. The size distribution was implemented using a Schulz distribution.^34^ The distribution was parametrized for both equatorial and polar radii using *z* = 5.6, *N*_pts_ = 80, and *N*_σ_ = 8, where *z* is the width of the distribution, *N*_pts_ is the number of points used to compute the function, and *N*_σ_ defines how far into the tails the distribution is considered in the calculation. This gives a polydispersity value of 0.15, which is a common value for structural fluctuations in globular micelles.^33^ Small variations of this parameter (±0.05) did not show significant variations in the results from the fits.

As the morphology of dodecyltrimethylammonium halide micelles remains rather unchanged with surfactant concentration in 1:2 choline chloride:glycerol,^29^ the experimental structure factor can be deconvoluted from the data using the known form factor (from, e.g., particle structure in the dilute regime) using eq 2:
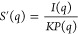
2where *K* is a factor that accounts for the particle concentration and contrast factor. As such, the experimental structure factor can be extracted and fitted using mathematical models that describe the interactions between particles.

The structure factor for spherical particles, *S*(*q*), has been analytically derived for different interaction potentials. The hard sphere (HS) model accounts for the correlation between particles interacting through excluded volume effects. This model is described using two parameters: the effective radius of the particle, *r*_eff_, and the volume fraction of particles, *ϕ*_p_, with a resulting number density, *n*, that matches that of the micelles and defined as
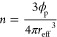
3

The interaction potential, *v*(*r*), at an interparticle distance *r* is calculated using the following closure relation to resolve the Ornstein–Zernike equation, where *r* is the center-to-center distance between particles:^18,35^
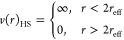
4

This model has been successfully applied to account for the interaction between uncharged particles or those with screened electrostatic interactions.^36,37^

The interaction component in the scattering for charged colloidal particles can be calculated using the rescaled mean spherical approximation (RMSA), a rework of the original mean spherical approximation derived by Hayter and Penfold to describe interactions at low particle volume fractions using the Yukawa potential.^19,20^ This model describes electrostatic interactions between charged hard spheres in an electrolyte solution of ionic strength *I* and uses the following closure relationship to calculate the interaction potential from the Ornstein–Zernike equation:
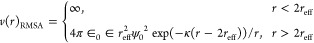
5where *ε*_0_ and ε are the vacuum permittivity and solvent relative permittivity, *k*_B_ is the Boltzmann constant, *T* is the system temperature, *ψ*_0_ is the particle surface potential, and κ is the inverse of the Debye screening length, which is given by eq 6:
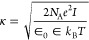
6where *N*_*A*_ is Avogadro’s number, *e* is the electron charge, and *I* is the ionic strength of the solution. Using the macroion charge, *z*_p_, the surface potential is calculated as
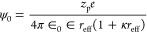
7

The reduced potential for the mean interparticle spacing, *a*, allows for determination of the Coulomb coupling constant, *Γ*_*k*_, from the fitting results using the eq 8:
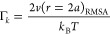
8And the mean interparticle spacing is approximated using^19^
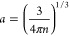
9which combined with eq 3 gives
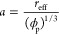
10

Note that the surface-to-surface distance between particles is given by *a* – 2*r*_eff_. The dimensionless Coulomb coupling constant, *Γ*_*k*_, relates to the strength of the electrostatic force exerted between particles separated by an arbitrary interparticle distance, *r*. This interparticle distance is conveniently defined as *r* = 2*a* and can be used to quantify electrostatic interactions within a colloidal dispersion. Further details in the derivation of the interaction potential can be found in the original reports.^19,20^

The *S*(*q*) models presented above (eqs 4 and 5) are derived for interacting spherical particles. However, the interpretation of scattering data from nonspherical particles is complicated by the shape- and orientation-dependent interaction, thus, resulting in inaccurate *S*(*q*) models for anisotropic particles. The decoupling approximation (DA) was developed to correct the scattering contribution from the interaction of nonspherical and polydisperse particles.^34^ This approach assumes that there is no correlation between particle position and orientation, and it has been proved a reasonable approximation for moderately anisotropic and polydisperse particles.^38,39^ The apparent interparticle structure factor is formally defined as
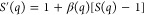
11
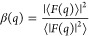
12where *F*(*q*) is the amplitude of the form factor and *S*(*q*) is the analytical structure factor for interacting spheres. The effective radius used to calculate the *S*(*q*) is defined as the radius of a spherical particle with the same second virial coefficient as the colloidal particles. This is determined for ellipsoids using eq 13:
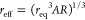
13

A systematic approach has been followed to analyze the SANS data presented here. Initially, the micelle form factor was determined from the micellar scattering in the dilute regime, and eq 2 was used to extract the experimental structure factor from higher surfactant concentrations. The parameters *r*_eff_ and *β(q)* were calculated from the fitted *P*(*q*) (eqs 12 and 13) and those were fixed during the analysis of the *S*′(*q*) data. The Coulomb coupling factor was used to parametrize the apparent structure factor models using the RMSA. Due to the correlation of the Debye length, of which the ionic strength of the solvent is unknown, and the particle surface potential (eq 5, 6 and 7), no specific values of the ionic strength or particle charge could be directly calculated. The results from the modeling of *S*′(*q*) were subsequently convoluted with the particle form factor, and the resulting *P*(*q*)·*S*′(*q*) models were validated against the experimental data.

Model-based analysis of the SANS data was performed using SasView 5.0.3, and the experimental data were fitted using the nonlinear least-squares method Levenberg–Marquardt algorithm. For further information on the definition of the mathematical models and functions, refer to the original references and the SasView documentation.^40^

## Results

**Figure 1:**
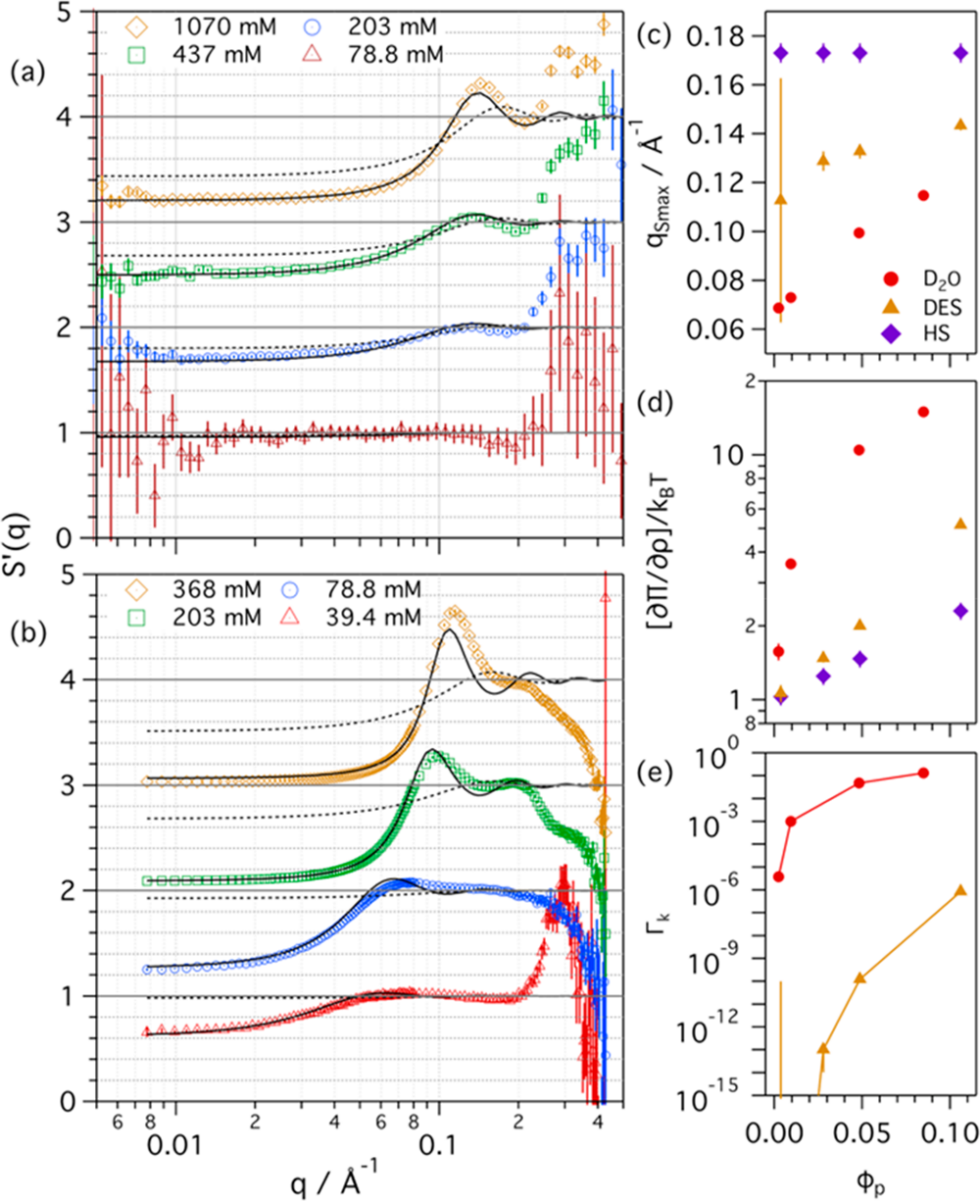
*S*(*q*)′ data and best fits from different concentrations of h-C_12_TAC micelles in (a) 1:2 d-ChCl:d-Glyc and (b) D_2_O. The concentrations of surfactant are presented in the legend of graph (a) and (b). Data were modeled using the HS model (dotted lines) and RMSA model (solid lines). Data and models were scaled for clarity. Variation of (c) *S*′(*q*) first peak position, (d) osmotic compressibility, and (e) Coulomb coupling constant as a function of micelle volume fraction in DES and D_2_O, and for HS interactions, as shown in the legend of graph (c). Where not seen, the error bars are within the markers.

The scattering of h-C_12_TAC in 1:2 d-ChCl:d-Glyc was initially measured at different surfactant concentrations, and these concentrations were decided based on previous investigations. The lowest concentration (78.8 mM) was selected to be in the dilute regime, where interparticle scattering is negligible, while still giving a good signal-to-noise ratio.^29^ The concentrated samples were measured up to a concentrated micellar phase (1070 mM), defined to be below the transition to lyotropic phases.^41,42^ As the form factor of the micelles remains unchanged in the concentration range investigated here (See Figure S1 and Table S1), the apparent structure factor, *S*′(*q*), was deconvoluted. The scattering from different concentrations of C_12_TAC in D_2_O was measured and treated using the same approach. The deconvoluted *S*′(*q*) and best fits using the RMSA model are presented in Figure 1, together with the main results from the analysis.^19,43^ The fits to the experimental scattering data are included in Figure S1, and the results from the analysis are presented in Table S1. The relative static permittivities used for the structure factor calculations are *ε*_DES_ = 22.8 and *ε*_D2O_ = 77.9.^44,45^

Initially, the experimental interparticle scattering was fitted using the HS model, which considers that interparticle interactions are limited to the excluded volume effects (no electrostatics). This approach showed poor agreement between the model and the experimental data, where the calculated intensities and oscillations in the model are far from those in the experimental data. Thus, excluded volume effects from the particle volume are not sufficient to account for the interparticle scattering. Subsequently, the experimental data were fitted using the RMSA model that accounts for electrostatic interactions between colloidal particles.^19,21^ As the definition of the structure factor as a function of *q* implies that *S*(*q*) = 1 for *q* → ∞, it should be noted that the lack of agreement for all models for *q* > ∼0.25 Å^–1^ is attributed to issues with the experimental *S*(*q*)′ deconvolution process (e.g., background subtraction) and only appears in the high-*q* data (see Figure S1). Thus, these are assumed to not affect the interpretation of the interparticle interactions from the *S*(*q*)′ data. The models obtained through this approach successfully describe the experimental data for the surfactant in both DES and D_2_O, as seen in Figure 1.

An initial comparison between the interactions observed in DES to those in water and those from the HS theoretical predictions can be made by looking at the position of the first peak in the *S*′(*q*) data, *q*_Smax_ (Figure 1c). This approach is not biased by any modeling assumptions. As the position of the peak in reciprocal space relates to the characteristic distance of correlated particle pairs in real space, lower *q*-values relate to longer interaction distances. Three initial observations can be made from these results: (1) the position of *q*_Smax_ changes with concentration for interparticle interactions in DES and water, but only small changes are observed in the position of the HS interaction peak; (2) the peak position goes to higher *q*-values when increasing particle volume fraction in DES and water; and (3) the *q*-values in DES are higher than those in water but lower than those from HS predictions. Unlike HS repulsion, the interaction distance between the particles in DES and water must go beyond the characteristic distance of the excluded volume, as observed in the shift of *q*_Smax_ to lower *q*-values. Thus, some long-range interactions are present in the systems and the interaction distance appears to be shorter in DES than in D_2_O.

From the fitted extrapolated structure factor at zero angle, *S*(0), the osmotic compressibility for each system can be calculated using the results from the analysis of the scattering data.
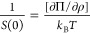
14And these values can be compared with the osmotic compressibility for hard spheres, which are theoretically predicted using eq 15:^46^
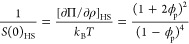
15

Figure 1d shows the calculated *S*(0) values and a comparison to the HS theoretical predictions. As expected, an increase in particle volume fraction leads to an increase in the osmotic compressibility for all the systems. The results show that the predictions made by the HS approach are far from the experimental compressibility in DES and water, and this difference increases at larger volume fractions. Also, the trend in the compressibility with ϕ_p_ in DES is similar to that in water but the absolute values are lower in DES at equivalent particle volume fractions. This again suggests that long-range interactions act upon both systems, but those are weaker in DES.

The observed long-range effects could be attributed to the presence of a strongly correlated shell of solvent components around the micelle (see SI, Figure S3 and Table S4). In this case, the solvent shell around the particles would be invisible to neutrons in terms of particle form factor. However, the presence of this shell would create an excluded volume effect that affects the structure factor contribution to the scattering. To probe this, the experimental structure factor of C_12_TAC in DES was fitted to an HS model where the effective radius, *r*_eff_, and effective volume fraction, ϕ_p,eff_, are not constrained to the particle form factor and volume fraction. Thus, these parameters can adopt any arbitrary values that somehow relate to the interparticle interaction. The same analysis was performed for C_12_TAC in D_2_O for comparison. The results show that the fits from this approach are relatively close to the experimental data. This is not surprising as an ersatz excluded volume effect has been previously used to approximate the scattering contribution from long-range interparticle interactions.^29,47^ However, different *r*_eff_ are required to acceptably fit the experimental *S*′(*q*) data for different concentrations of surfactant in both DES and aqueous solution. Also, the ϕ_p,eff_ are considerably higher than those of the particles, ϕ_p_.

Electrostatic interactions between particles can be parametrized through the Coulomb coupling constant, Γ_k_. This parameter directly relates to the strength of the interaction potential at the average interparticle distance. Changes in Γ_k_ for the micelles in DES and water as a function of micelle volume fraction are shown in Figure 1e. It should be noted that this parameter is defined as zero for HS interactions. As with the changes observed in water, the coupling constant increases with micelle volume fraction in DES. The *S*′(*q*) effects at low particle volume fraction in DES are very weak, and thus, the uncertainties in the fitted parameters are very large. This value becomes better defined at higher volume fractions but is still several orders of magnitude below the coupling constant for the interactions that occur in water. Therefore, these results confirm that long-range interactions occur in DES, probably electrostatic in nature, and those are weaker in the DES than the repulsion between micelles in water.

**Figure 2:**
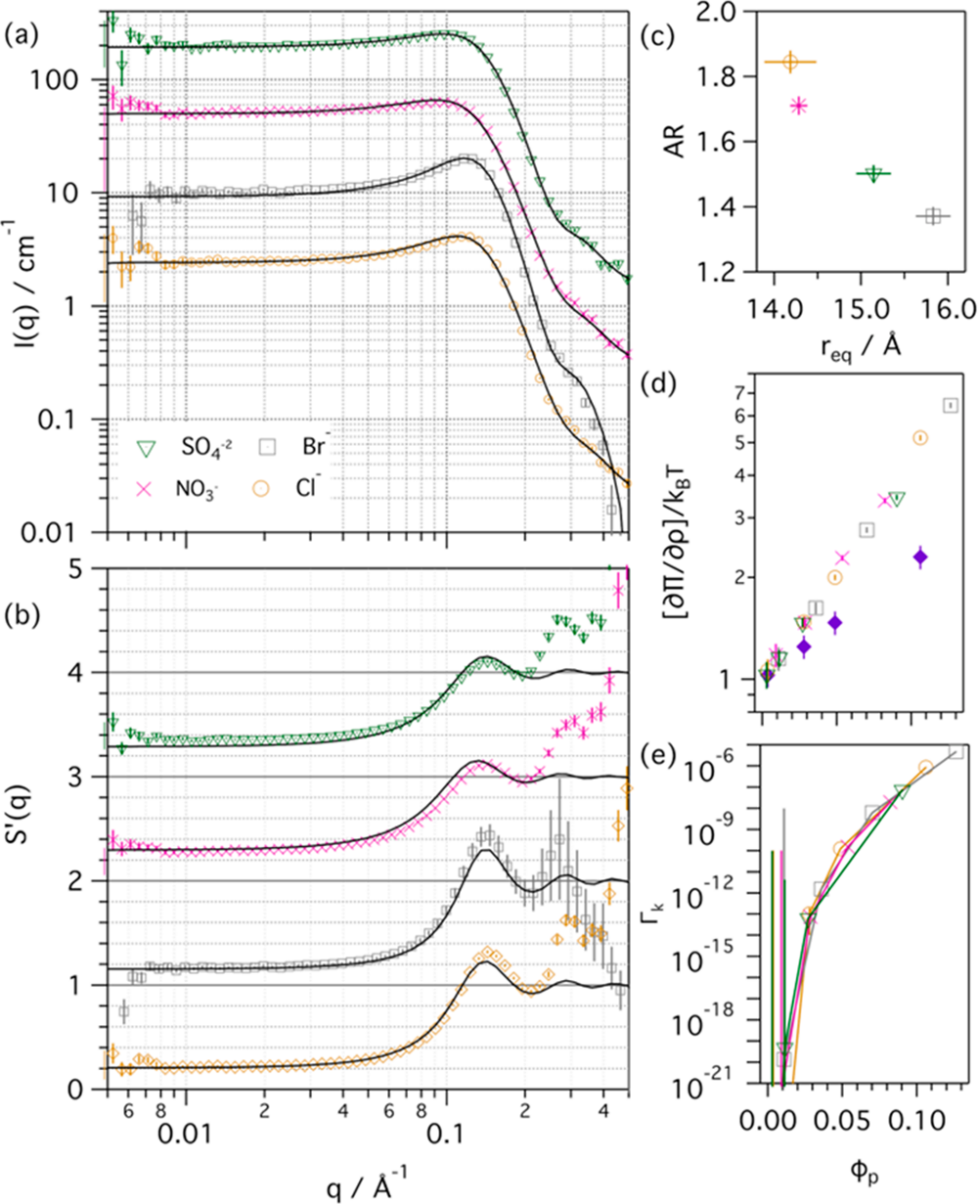
(a) SANS data and (b) *S*(*q*)′ data of counterion-exchanged 966 mM (average concentration) h-C_12_TA^+^ micelles in 1:2 d-ChCl:d-Glyc, as shown in the legend of graph (a). The experimental data were modeled (solid lines) using a uniform ellipsoid form factor and a RMSA structure factor. Data and models were scaled for clarity. Variation of (c) micelle structural parameters *r*_eq_ and AR, (d) osmotic compressibility, and (e) Coulomb coupling constant as a function of micelle volume faction for each counterion-exchanged surfactant in DES. These results are compared to the theoretical predictions from HS interactions (purple solid markers). Where not seen, the error bars are within the markers.

Another important effect that governs macroion interactions in colloidal systems is ion condensation and specific ion effects at the particle interface. The experimental scattering data and the apparent structure factor of different counterion-exchanged C_12_TA^+^ surfactants in 1:2 d-ChCl:d-Glyc are presented in Figure 2. The fits to the experimental scattering data and results from other surfactant concentrations are included in Figure S2 and Table S2.

As the salt concentration in the DES (ca. 2.5 M) is significantly higher than that of the surfactant native counterion (from 78.8 to 1070 mM), it could be expected that specific ion effects vanish in this environment. However, this is not the case, as micelle morphology is affected by changes in the surfactant counterion despite the dominant concentration of solvent ions. The structural parameters extracted from modeling the form factor show that both *r*_eq_ and AR vary between the different surfactant counterions, while different concentrations of each surfactant were satisfactorily fitted using the same form factor (see Figure 2c, Figure S2 and Table S2). Also, the fitted micelle volume fractions in DES are considerably lower than the expected values when neglecting monomer content. Considering the total surfactant concentration and the fitted micelle volume fraction, the volume fraction and concentration of surfactant monomers (*ϕ*_m_ and [C_12_TA^+^]_m_, respectively) in solution were calculated as described in the SI. The results are presented in Table 2 for a single surfactant concentration, and a full record of the calculated values for all surfactant concentrations is presented in Table S5. It should be noted that the monomer concentration is only equal to the critical micelle concentration (CMC) at the CMC, and it can evolve in either direction above this threshold concentration in aqueous solution.^48^

**Table 2:** Calculated Monomer Volume Fraction and Concentration of Monomer for Different Surfactant Counterions of h-C_12_TA^+^ in 1:2 d-ChCl:d-Glyc: SO_4_^–2^, Br^–^, NO_3_^–^, and Cl^–^

Surfactant	[C_12_TA^+^]/mM	*ϕ*_s_/×10^–2^	*ϕ*_p_/×10^–2^	*ϕ*_m_/×10^–2^	[C_12_TA^+^]_m_/mM
h-C_12_TAC	78.8	2.04	0.35 ± 0.02	1.69 ± 0.02	57.3 ± 0.7
h-C_12_TAB	79.7	2.43	1.08 ± 0.03	1.34 ± 0.03	45.7 ± 1.0
h-C_12_TANO_3_	83.9	2.38	0.90 ± 0.02	1.48 ± 0.02	50.3 ± 0.7
h-C_12_TA(SO_4_)_1/2_	85.8	2.32	0.29 ± 0.04	2.01 ± 0.04	69.1 ± 1.4

The results from the calculations reveal that there is a high concentration of dissolved monomer in solution, and it is counterion dependent. The variation of surfactant solubility follows the trend [C_12_TAB]_m_ < [C_12_TANO_3_]_m_ < [C_12_TAC]_m_ < [C_12_TA(SO_4_)_1/2_]_m_. Interestingly, the concentration of surfactant monomer increases for all surfactants when the total surfactant concentration is increased (see Table S5), proving that the surfactant monomer concentration is not constant above the CMC.

The long-range interactions of counterion-exchanged surfactant micelles were also probed using these data (Figure 2b). For all these surfactants in DES, it is seen that the osmotic compressibility is higher than those values from the HS theoretical predictions and these excess interactions are hypothesized again to be of electrostatic origin. When comparing the osmotic compressibility for the different surfactants (Figure 2d), the results follow a similar trend and only small differences are observed. Also, the results for the Coulomb coupling constant follow the same trends and only subtle differences between the various counterion-exchanged surfactants are observed. To directly compare the strength of the electrostatic interactions, the experimental coupling constants were modeled using a simple empirical approach and the coupling constants of the different systems were calculated for a theoretical volume fraction of 10% using those models (see SI, Figure S4, Table S6). Among the counterions investigated here, it is observed that the coupling constant for *ϕ*_p_ = 0.1 of h-C_12_TAB and h-C_12_TANO_3_ in 1:2 d-ChCl:d-Glyc are 31% (±5%) and 56% (±8%) lower than that for h-C_12_TAC, respectively. On the contrary, the coupling constant for h-C_12_TA(SO_4_)_1/2_ at the same volume fraction is 13% (±4%) higher than that for h-C_12_TAC. These observations confirm that the strength of the intermicellar interactions depends on the surfactant counterion, where it follows the trend, from weaker to stronger, NO_3_^–^ < Br^–^ < Cl^–^ < O_4_^–2^. As such, the specific ion effects also modulate long-range interparticle interactions in the DES.

**Figure 3:**
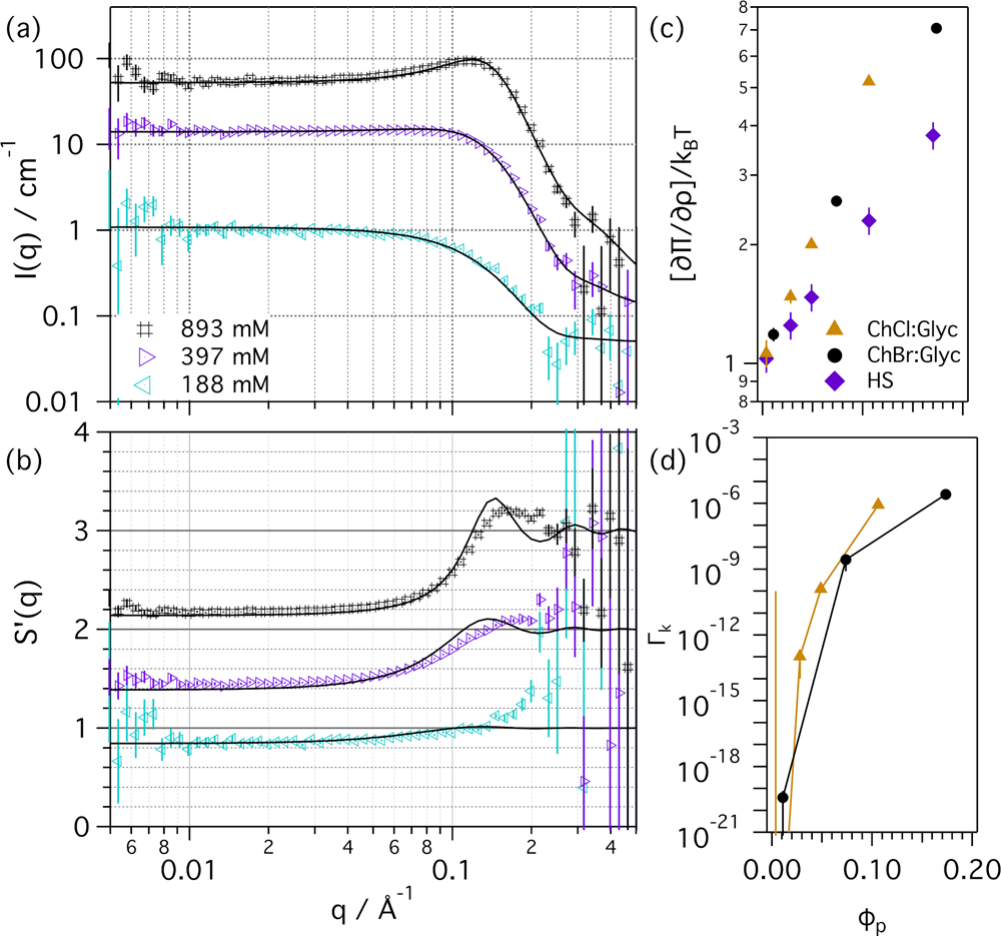
(a) SANS data and (b) *S*(*q*)′ data from different concentrations of d-C_12_TAB micelles in 1:2 h-ChBr:h-Glyc, as shown in the legend of graph (a). The experimental data were modeled (solid lines) using a uniform ellipsoid form factor and an RMSA structure factor. Data and models were scaled for clarity. Variation of (c) osmotic compressibility and (d) Coulomb coupling constant as a function of micelle volume faction for d-C_12_TAB micelles in 1:2 h-ChBr:h-Glyc and h-C_12_TAC micelles in 1:2 d-ChCl:d-Glyc, and for HS interactions, as shown in the legend of graph (c). Where not seen, the error bars are within the markers.

Due to the ionic nature of the DES, it is hypothesized that the resulting ion-pair interactions rely on a balance between the interactions of the surfactant counterion with either the micelle or the ionic species in the bulk solvent. To prove this, we have investigated the self-assembly of d-C_12_TAB in 1:2 h-ChBr:h-Glyc and compared that to the behavior of h-C_12_TAC in 1:2 d-ChCl:d-Glyc. This study provides an insight into the role of the main ions involved in the condensation of counterions at the interface of the cationic micelles. The experimental scattering data and models for d-C_12_TAB in 1:2 h-ChBr:h-Glyc are presented in Figure 3. The results from the analysis are presented in Table S3.

It is important to note that, due to the lack of data on the physicochemical properties of the bromide-based DES, those values were approximated as those of the chloride-based DES for data analysis purposes. When comparing the results from the data analysis, small differences in micelle structure were found between the two systems (see Tables S1 and S3). The calculated osmotic compressibility of d-C_12_TAB micelles in 1:2 h-ChBr:h-Glyc increases with increasing micelle volume fraction (see Figure 3c), as observed for the system in the chloride-based DES. However, these values are lower than those for h-C_12_TAC micelles in 1:2 d-ChCl:d-Glyc and again higher than those from HS predictions. When comparing the values from the Coulomb coupling constant (see Figure 3d), it is seen that the strength of the intermicellar interactions in 1:2 h-ChBr:h-Glyc is slightly lower than that in 1:2 d-ChCl:d-Glyc, in line with the osmotic compressibility results. Therefore, electrostatic interactions between micelles are weaker in the bromide-based DES than in the chloride analogue.

## Discussion

### Long-Range Electrostatic Colloidal Interactions

The interactions attributed to HS packing, commonly used to fit the scattering from uncharged or screened micelles,^37^ are not appropriate to describe the behavior of h-C_12_TAC micelles in either d-DES or D_2_O, and an excess interaction seems to act upon the system. When attempting to fit the intermicellar structure factor using the RMSA model, which describes long-range electrostatic interactions between colloidal particles, good agreement between data and models was found. This agreement suggests that the excess interparticle interaction in DES may be attributed to the Coulombic repulsion between the macroions in solution, as it occurs in water.^49^ The main difference observed in the interactions between h-C_12_TAC micelles in d-DES or D_2_O is that those are weaker in the case of the DES, suggesting that the electrostatic interactions are partially screened in DES compared to those in water. Another model that could be considered to describe the long-range effects in DES is that resulting from the excluded volume between strongly correlated solvent shells around the micelles. From the analysis of the experimental structure factor using an effective excluded volume to account for this shell (see SI, Figure S3, Table S4), it is observed that the effective radius varies with surfactant concentration for the C_12_TAC micelles in DES and aqueous solution. As the solvation of the particle is not expected to change with the C_12_TAC concentration, the *r*_eff_, i.e. the “hard” solvation shell around the particle, should remain constant if the long-range interactions were solely attributed to the overlap between these shells. As this value changes with surfactant concentration, the long-range interactions cannot be attributed to the exclusion between correlated solvent shells around the micelle.

One of the main contributions to the differences in the strength of the interaction between these two solvents must arise from differences in the permittivity and ionic strength of the continuum, parametrized here as the Debye length. In order to rationalize the difference in the strength of the interaction, we first consider a thought experiment: Assuming the same arbitrary ionic strength for the two solvents (e.g., 20 mM), the differences in the calculated Debye lengths will only depend on the solvent permittivity (*ε*_DES_ = 22.8 and *ε*_water_ = 77.9). As such, the Debye lengths in this hypothetical case are ∼12 Å and ∼22 Å for DES and water, respectively, which is a ca. 45% difference in the Debye length between these two electrolytes. When estimating the Coulomb coupling constant for an arbitrary particle charge (e.g., 30), the difference in the values between these two solvents becomes ca. 450%. However, the difference in the Coulomb coupling constant from the analysis of the scattering data is around 6 orders of magnitude larger in water than in DES. As the permittivity of the solvents does not suffice to account for the difference in the coupling constant, there must also be a significant contribution from the ionic strength of the continuum.

From theoretical calculations, it is seen that the coupling constant rapidly decays when increasing the ionic strength of the solvent as interactions at the average interparticle distance become weaker (see SI, Table S7, Figure S5). However, the coupling constant values in DES are still above zero, showing that excluded volume effects without long-range effects are not sufficient to account for the interparticle interactions. Therefore, the apparent ionic strength of the DES must be much higher than that of D_2_O, as could be expected. Due to the mathematical correlation between the solvent ionic strength and particle charge in the calculation of the surface potential and Coulomb coupling constant (eqs 5, 6, and 7), these parameters could not be accurately calculated. To shed some light on how these vary for the fitted coupling constant, a sensitivity analysis was performed (see SI, Table S8). From this analysis, it is observed that the effective ionic strength of the solvent falls between ca. 340 and 540 mM for a realistic range of macroion charges (assuming counterion dissociations between 20% and 80%). This confirms that the effective ionic strength of the DES is significantly lower than the theoretical ionic strength of this solvent (ca. 2.5 M, as calculated from the complete dissociation of choline chloride), which in turn would lead to negligible Debye lengths (ca. 1.5 Å) and the absence of long-range electrostatic interactions. Consequently, the Debye length of the DES appears to be considerably larger than the theoretical Debye length for this solvent, as some partially screened electrostatic interactions between particles prevail in DES. One of the remaining challenges to quantify the effects of electrostatic interactions and counterion condensation (e.g., particle surface charge) is to accurately characterize the ionic character of the DES (e.g., Debye length). This will provide a better understanding of the fundamental effects presented here and will enable direct comparison to other highly ionic systems.

Considering these results, parallels can be drawn with the behavior of concentrated electrolytes and ionic liquids. As it has recently been shown for long-range electrostatics in systems with a high ion concentration, the apparent screening length is much larger than that theoretically predicted by the Debye–Hückel theory.^8,9,50^ This effect has been associated with the strong ion–ion correlations within the bulk phase in concentrated ionic environments. As such, the bulk liquid is mainly constituted by strongly correlated ions that do not effectively contribute to the ionic strength of the solvent, while a relatively small number of thermally excited ions can act as charge carriers.^51^ Thus, the resulting screening effect is much weaker than that for noncorrelated ions and the system behaves as a relatively dilute electrolyte. We hypothesized here that an analogous effect is observed in DES: ion-pair correlations within the bulk reduce the apparent ionic strength of the solvent and are the mechanistic origin of the long-range electrostatic interactions in DES. In the case of DES, it could be expected that the system behaves in a similar way to highly concentrated ionic solutions in neutral solvents.^52^ The neutral moiety of the DES would contribute to the partial disruption of ion-pair correlations and allow a certain population of free ions.

The ion–ion correlation in DES has also been evidenced by other investigations. For example, the ion conductivity decreased in neat DES compared to hydrated DES, in stark contrast to dilute electrolytes that increase conductivity with increasing salt content.^53^ As the salt concentration in the DES components increases with decreasing hydration level, ion mobility is hindered by molecular interactions between the solvent constituents, resulting in a strong ion–ion correlation and low conductivity. Also, it has been shown that the charged surface of colloidal particles induces fluctuations in the structure of the solvent and the formation of multilayer perturbations, possibly attributed to electrostatic interactions that extend beyond the nanometer scale.^54,55^ Similarly, the nanostructure of the solvent was found to be affected up to a few nanometers at the platinum–DES interface with applied potential.^56^ Molecular dynamic simulations showed that charge spreading occurs through the DES network, resulting in an effective charge density lower than that of the compounds in an ideal gas phase.^57^ Thus, the hypothesis of the prevalence of long-range electrostatic effects in DES as the result of strong ion–ion correlation in the continuum is further supported by previous studies using a variety of methods.

### Specific Ion Effects

The condensation of ions at the micelle interface, either the native surfactant counterion or from added salts, is a well-known effect in aqueous solution, where this modulates electrostatic interactions, affects monomer solubility, and results in changes in micelle morphology.^58−61^ Here, we show that different surfactant counterions lead to variations in micelle structure in the DES, as the radius and aspect ratio of the micelle changes. Also, the monomer solubility changes with varying the counterion and volume fraction of surfactant. These effects have previously been reported for anionic surfactant micelles in DES, where the substitution of the counterion in dodecylsulfate surfactant solutions resulted in changes in micelle morphology and CMC.^62^ Similarly, other specific charge interactions with hydrotropes and solvent ions have been shown to affect the morphology of micelles in DES.^47,63−65^

The relatively high concentration of surfactant monomers could relate to the weaker solvophobic effect in DES compared to that in water.^66^ For instance, the CMC of C_12_TAB in 1:2 choline chloride:glycerol (CMC = 22 mM) has been shown to be higher than those in aqueous solution (CMC = 15 mM), but significantly lower than that in ethylammonium nitrate (190 mM).^29,67,68^ The weaker solvophobic effect also results in a higher monomer concentration in the micellar phase, and the solubilized surfactant monomers in 1:2 choline chloride:glycerol could deviate the solvent from its native composition and structure. Previous investigations have shown that an imbalance in the eutectic composition affects the molecular behavior of DES.^69^ However, the presence of a neutrally charged graphite interface, which could cause similar effects in the solvent structure to those induced by the hydrophobic surfactant tails, only prompts subtle short-range changes in the molecular ordering of 1:2 choline chloride:glycerol.^70^ At the surfactant concentrations investigated here, these local changes are not expected to alter the bulk behavior of the DES, as correlations between the solvent components are relatively resilient.^11,71^ Also, the trends observed in intermicellar interactions and the unchanged micelle morphology in the concentration range investigated here suggest that no significant changes in the behavior of the solvent occur when increasing the surfactant content. However, further investigations are required to probe the presence of specific surfactant–DES interactions at the molecular level.

Specific ion effects have also been reported for the micellization of surfactants in aqueous electrolytes and ionic liquids. Small-angle neutron scattering was used to demonstrate that the adsorption of surfactant counterions controls the micellization in protic ionic liquids, affecting the structure of the globular micelles due to specific counterion condensation.^72^ Interestingly, whereas C_12_TA^+^ micelles in water increase in aggregation number with addition of salt which results in the formation of elongated micelles,^73^ these remain globular in DES and ionic liquids despite the high ionic strength of the solvent.^72^ Also, the solubility of surfactant monomer has been shown to vary in ionic liquids depending on the surfactant counterion.^72^ The observed variation of surfactant monomer concentration above the CMC is an effect that has previously been reported for C_12_TAB in aqueous solution, where the monomer concentration gradually decreases above the CMC with increasing surfactant concentrations.^48,74^ However, it is observed that the content of solubilized monomer above the CMC follows the opposite trend in DES. The mechanistic origin of this stark difference is however unknown, and there must be a deeper meaning related to the surfactant activity and monomer–micelle equilibrium.

The effect of counterion exchange is also shown to modulate electrostatic interactions between particles in DES, as previously shown for aqueous solutions of micelles.^61,75^ It is hypothesized that certain ions are more prone to condense at the micelle interface and screen interparticle interactions to a larger extent in DES, following a similar mechanism to that in water. The condensation of counterions will potentially result in a change in the surface potential of the micelle, which is the main source for the differences in the coupling constants between the different counterion-exchanged surfactant systems, as the ionic strength of the continuum will remain relatively unchanged. Interestingly, the strength of the interaction follows the same order as it does in water (from weaker to stronger, NO_3_^–^ < Br^–^ < Cl^–^ < SO_4_^–2^).^75^ These specific condensation effects have also been observed for colloidal particles in DES, where exogenous Ag^+^ ions were found to preferentially populate regions around SiO_2_ particles, demonstrating that specific ion–ion interactions occur to the detriment of choline–particle interactions.^54^

These specific ion effects are attributed to the energetic balance between the ion in a solvated state and that in a condensed state, ultimately leading to counterion condensation. This balance differs depending on the character of the ion and have traditionally been described by the Hofmeister or lyotropic series in aqueous solutions, ionic liquids, and other nonaqueous solvents.^75−77^ For instance, it is expected that a bromide counterion will interact more strongly with a cationic micelle than chloride, which in turn will remain more solvated and result in a more pronounced electrostatic effect. This is a similar behavior to that observed in the case of the cationic micelles investigated here, where the coupling constants for the bromide and nitrate counterions are lower than those for the chloride and sulfate counterions. It is expected that the trends in terms of counterion solubility follow the opposite order: NO_3_^–^ < Br^–^ < Cl^–^ < SO_4_^–2^. Therefore, the Hofmeister series can describe specific ion effects in DES.

It has recently been shown that electronic perturbations as induced by ion pairs and ion–solvent interactions are the underlying phenomena that control ion solvation and condensation effects in aqueous and nonaqueous molecular solvents.^78^ These findings could also be used to describe the specific ion effects in DES. Unlike in aqueous solution where the ion effects can simply be rationalized in terms of the interaction between a pair of ionic species,^75^ these effects in DES are far more complex due to the ionic character of the solvent. As the ion-pair interaction relies on a balance between the counterion–headgroup interactions (i.e., ion condensation free energy) and the counterion–solvent interactions (i.e., ion solvation free energy), the resulting counterion condensation will be defined by the interplay between these two ionic perturbations. This has been shown for the self-assembly of surfactants in ternary DES, where the solubility of the counterion modulates micelle morphology.^65^ There, the reduction of the solubility was shifting the energetic balance toward the condensed state at the micelle interface. This was investigated here by comparing the long-range interactions in the counterion-exchanged solvents. As the surfactant counterion is the same as the solvent anion (Br^–^ in 1:2 h-ChBr:h-Glyc, Cl^–^ in 1:2 d-ChCl:d-Glyc), no specific ion–solvent effects are expected for these systems. As such, the differences in the electrostatics are mainly attributed to the dissociation of surfactant counterions in each solvent and the permittivity of those (here assumed to be the same for analysis purposes). The results showed that stronger electrostatic interactions appear in the case of the h-C_12_TAC solution in 1:2 d-ChCl:d-Glyc in comparison to the d-C_12_TAB solution in 1:2 h-ChBr:h-Glyc. This shows that the association of Cl^–^ counterions with the cationic micelles is weaker than that of Br^–^, thus confirming the specificity of the counterion condensation.

## Conclusions

Deep eutectic solvents are promising sustainable alternatives to traditional solvents in a vast array of technological products and processes. From mimicking biological environments in the absence of water for biomolecule preservation to the development of cheap and green electrolytes, understanding the fundamental phenomena that govern electrostatic interactions in DES opens new avenues for research. Here, we presented the first investigation of electrostatic interactions in DES. Small-angle neutron scattering was used to probe particle structure and particle–particle correlation in micellar systems dispersed in 1:2 choline chloride:glycerol and 1:2 choline bromide:glycerol.

The results show that long-range interactions prevail in DES and are electrostatic in origin. These interactions are weaker than those for the analogous system in aqueous solution, and this was attributed to the higher ionic strength of the DES. However, this effective ionic strength seems to be much lower than the actual ion concentration in the DES (*ca*. 2.5 M), which would lead to negligible long-range electrostatics. The mechanistic origin of these electrostatic interactions is hypothesized to arise from the strong ion-pair correlation in DES, where strong short-range correlations between ions within the solvent reduce the apparent ionic strength in DES. This parallels recent theories on the behavior of charge interactions in ionic liquids and concentrated electrolytes.

Also, specific ion effects, which play a key role in colloidal stability and protein behavior, are demonstrated to modulate micelle morphology, surfactant solubility, and long-range electrostatic interactions in DES. These effects were attributed to the counterion condensation at the micelle interface, and the Hofmeister series can be used to describe the observed trends. These interactions are classically described as a single ion pair in water, where the free energy of solvation/condensation controls the extent of the adsorption. However, the mechanism seems to be more complex in DES, where electrostatic correlations within the solvent potentially affect the interactions of the counterion with the surfactant.

As such, DES join the group of solvents with high ion concentrations (i.e., concentrated electrolytes and ionic liquids) that challenge the traditional understanding of electrostatic interactions. Therefore, the investigations presented here will have diverse implications in fundamental and applied research. For instance, a better understanding of the electrostatic and ion-specific effects in DES will influence the development of new colloidal systems, the design of novel environments for the stabilization and function of biomolecules, and the preparation of sustainable electrolytes using DES. Also, these results contribute to the development of a benchmark theory that describes electrostatic interactions in concentrated ionic environments.

## Supporting Information Available

A detailed description of the preparation of the counterion exchanged surfactants, a record of the SANS data modeling, the results from the calculation of the monomer concentration in DES, and the modeling approach of the Coulomb coupling constant. Data supporting this article has been made available at DOI: 10.5286/ISIS.E.RB1620126. (PDF)
