# Supplementary material for: Long-Range Electrostatic Colloidal Interactions and Specific Ion Effects in Deep Eutectic Solvents
Source: J Am Chem Soc. 2021 Aug 30;143(35):14158–68. doi: 10.1021/jacs.1c04781 (PMC8431340; doi:10.1021/jacs.1c04781)
Supplement: Supplementary file 1 — ja1c04781_si_001.pdf [file ja1c04781_si_001.pdf]

Supporting Information for:

Long-range electrostatic colloidal interactions and specific ion effects in deep eutectic solvents

Adrian Sanchez-Fernandez,[a]* Andrew J. Jackson,[b,c] Sylvain F. Prévost,[d] James J. Doutch,[e] Karen J. Edler[f]

[a]Food Technology, Engineering and Nutrition, Lund University, Box 124, 221 00 Lund, Sweden.

[b]European Spallation Source, Box 176, 221 00 Lund, Sweden.

[c]Department of Physical Chemistry, Lund University, Lund, SE-221 00, Sweden.

[d]Institut Laue-Langevin, 71 Avenue des Martyrs, 38000, Grenoble, France.

[e]ISIS Neutron and Muon Source, Science and Technology Facilities Council, Rutherford Appleton Laboratory, Didcot, OX11 0QX, UK.

[f]Department of Chemistry, University of Bath, Claverton Down, Bath, BA2 7AY, UK.

*E-mail: adrian.sanchez-fernandez@food.lth.se

Synthesis and characterization of the counterion-exchanged surfactant

The counterion-exchanged surfactants were prepared by exchanging the native chloride counterion from dodecyltrimethylammonium chloride ($C_{12}TAC$, Sigma-Aldrich, >99%) using a modification of the procedure previously reported for the cationic surfactants.[1] The ion exchange resin Amberlite IRN-78 hydroxide form (Sigma-Aldrich) was loaded into a chromatography column with a porous frit to retain the particle packing. The resin was initially washed using a 1 M NaOH solution and subsequently washed with abundant Milli-Q water to remove any excess base remanent in the column. Once the eluted solvent was neutral in pH, the column was ready for the counterion exchange.

$C_{12}TAC$ was dissolved in milli-Q water (*ca.* 800 mM) and loaded into the column at room temperature. The solution was subsequently pass through the column at least 5 times. The complete exchange was verified by the addition of a drop of silver nitrate solution to an aliquot of the surfactant solution. The exchange was completed when no AgCl precipitation could be observed, confirming the absence of any remanent chloride in the surfactant solution.

The resulting solution of dodecyltrimethylammonium hydroxide was neutralized to pH 7 using the corresponding acid solutions (1 M): HBr, HNO_3, or H_2SO_4. The neutralized solution was freeze-dried to remove the water and the powder was recovered for purification. The surfactants were dissolved and triple-recrystallized using ethanol/dry diethyl ether. Any residual solvent was removed *in vacuo*. The purity of the surfactants was assessed by ^{1}H-NMR.

Results from the SANS data analysis

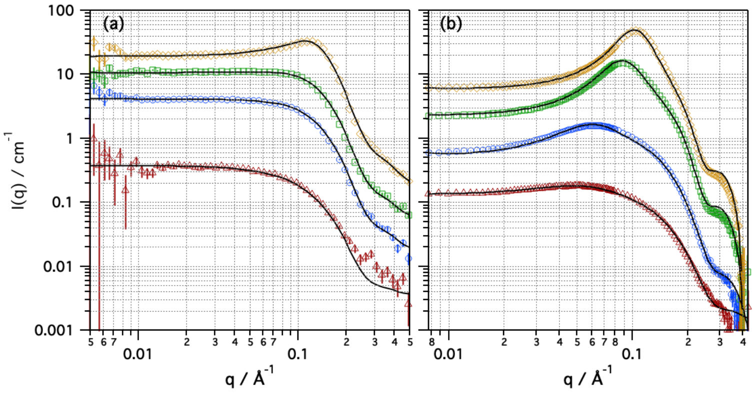

Figure S1 SANS data and best fits from different concentrations of h-$C_{12}TAC$ micelles in (a) 1:2 d-ChCl:d-Glyc (78.8 mM-△, 203 mM-○, 437 mM-□, and 1070 mM-◇) and (b) D_2O (39.4 mM-△, 78.8 mM-○, 203 mM-□, and 368 mM-◇). Data were modelled using the uniform ellipsoid P(q) and RMSA S(q) implemented using the DA. Data and models were scaled for clarity. Where not seen, the error bars are within the markers.

Table S1 Parameters obtained from the analysis of the SANS data presented in Figure S1: equatorial radius – r_{eq}, aspect ratio – AR, micelle volume fraction – ϕ, q-position of the first peak in the S(q) data – q_{Smax}, S(q) intensity at zero angle – S(0), and Coulomb coupling factor – Γ_k.

[$C_{12}TAC$] / mM	r_{eq} / Å	AR	ϕ_p / $\times10^{-2}$	q_{Smax} / Å^{-1}	S(0) / cm^{-1}	Γ_k
h-$C_{12}TAC$ in d-ChCl:d-Glyc						
78.8	14.3±0.3	1.71±0.06	0.35±0.03	0.113±0.053	0.942±0.075	$4.25\times10^{-31}\pm1.01\times10^{-10}$
203	14.3±0.3	1.71±0.06	2.79±0.04	0.129±0.004	0.677±0.027	$1.03\times10^{-13}\pm 9.28\times10^{-14}$
437	14.3±0.3	1.71±0.06	4.87±0.05	0.133±0.003	0.499±0.010	$1.24\times10^{-10}\pm1.64\times10^{-12}$
1070	14.3±0.3	1.71±0.06	10.6±0.1	0.143±0.002	0.193±0.004	$8.62\times10^{-7}\pm4.97\times10^{-9}$
h-$C_{12}TAC$ in D_2O						
39.4	14.1±0.2	1.49±0.06	0.23±0.03	0.069±0.002	0.637±0.012	$3.74\times10^{-6}\pm1.93\times10^{-8}$
78.8	15.1±0.2	1.45±0.06	0.93±0.03	0.073±0.002	0.279±0.006	$1.01\times10^{-3}\pm5.56\times10^{-5}$
203	16.5±0.1	1.33±0.04	4.82±0.02	0.099±0.001	0.096±0.002	$4.8\times10^{-2}\pm5.91\times10^{-4}$
368	16.8±0.1	1.31±0.04	8.49±0.02	0.115±0.001	0.067±0.001	$1.32\times10^{-1}\pm2.16\times10^{-4}$

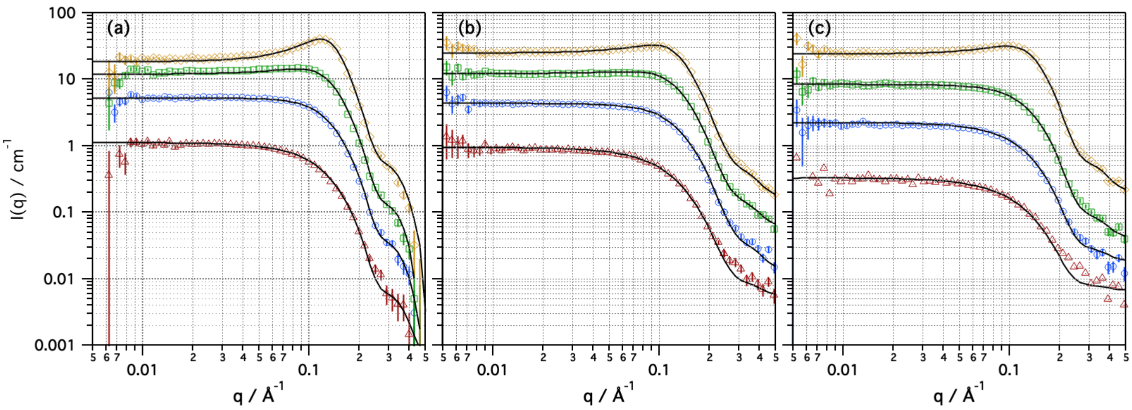

Figure S2 SANS data and best fits from (a) h-C_{12}TAB, (b) h-$C_{12}TANO_3$, and (c) h-$C_{12}TA(SO_4)_{1/2}$ micelles in 1:2 d-ChCl:d-Glyc at different surfactant (average) concentrations (78.8 mM-△, 203 mM-○, 437mM-□, and 1070 mM-◇). Data were modelled using the uniform ellipsoid P(q) and RMSA S(q) implemented using the DA. Data and models were scaled for clarity. Where not seen, the error bars are within the markers.

Table S2 Structural parameters obtained from the analysis of the SANS data presented in Figure S2.

$[C_{12}TA^+]$ / mM	r_{eq} / Å	AR	ϕ_p / ×10^{-2}	q_{Smax} / $Å^{-1}$	S(0) / cm^{-1}	Γ_k
h-C_{12}TAB in d-ChCl:d-Glyc						
79.7	15.8±0.3	1.38±0.08	1.08±0.03	0.092±0.073	0.868±0.069	$1.38\times10^{-20}\pm1.01\times10^{-8}$
205	15.8±0.3	1.38±0.08	3.60±0.05	0.125±0.008	0.616±0.025	$1.54\times10^{-12}\pm4.28\times10^{-20}$
422	15.8±0.3	1.38±0.08	7.01±0.08	0.136±0.006	0.362±0.007	$6.22\times10^{-9}\pm1.64\times10^{-15}$
943	15.8±0.3	1.38±0.08	12.6±0.1	0.146±0.002	0.155±0.003	$4.77\times10^{-6}\pm4.97\times10^{-9}$
h-$C_{12}TANO_3$ in d-ChCl:d-Glyc						
83.9	14.2±0.4	1.84±0.11	0.90±0.05	0.114±0.092	0.847±0.068	$3.43\times10^{-22}\pm1.10\times10^{-10}$
200	14.2±0.4	1.84±0.11	2.87±0.04	0.115±0.011	0.680±0.027	$7.42\times10^{-14}\pm1.28\times10^{-16}$
426	14.2±0.4	1.84±0.11	5.37±0.05	0.132±0.007	0.438±0.009	$1.54\times10^{-10}\pm1.64\times10^{-11}$
932	14.2±0.4	1.84±0.11	8.22±0.06	0.136±0.003	0.297±0.006	$1.93\times10^{-8}\pm1.47\times10^{-9}$
h-$C_{12}TA(SO_4)_{1/2}$ in d-ChCl:d-Glyc						
86.0	15.1±0.2	1.50±0.06	0.29±0.07	0.103±0.067	0.969±0.077	$1.09\times10^{-33}\pm1.11\times10^{-10}$
205	15.1±0.2	1.50±0.06	1.13±0.08	0.122±0.004	0.862±0.034	$4.78\times10^{-20}\pm5.13\times10^{-12}$
436	15.1±0.2	1.50±0.06	2.97±0.04	0.131±0.002	0.680±0.014	$6.30\times10^{-14}\pm1.34\times10^{-16}$
965	15.1±0.2	1.50±0.06	9.03±0.08	0.139±0.002	0.290±0.006	$7.27\times10^{-8}\pm3.82\times10^{-9}$

Table S3 Structural parameters obtained from the analysis of the SANS data of different concentrations of d-C_{12}TAB in h-ChBr:h-Glyc.

$[C_{12}TAB]$ / mM	r_{eq} / Å	AR	ϕ_p / ×10^{-2}	q_{Smax} / $Å^{-1}$	S(0) / cm^{-1}	Γ_k
d-C_{12}TAB in h-ChBr:h-Glyc						
B1	14.5±0.2	1.76±0.12	1.08±0.14	0.107±0.008	0.844±0.034	$3.85\times10^{-20}\pm6.42\times10^{-21}$
B2	14.5±0.2	1.79±0.08	7.37±0.06	0.148±0.003	0.388±0.008	$2.71\times10^{-9}\pm1.91\times10^{-9}$
B3	14.5±0.2	1.92±0.06	17.3±0.2	0.170±0.004	0.141±0.003	$2.55\times10^{-6}\pm2.55\times10^{-7}$

Excluded volume effects from a hard solvent shell

An alternative theoretical model that could result in long-range interactions in DES is the presence of a strongly correlated solvent shell around the micelle. In order to validate this model, the experimental S(q) of C_{12}TAC in d-ChCl:d-Glyc and in D_2O were fitted to an effective HS model, as presented in the main text. The results from this analysis are presented in Figure S3 and Table S4.

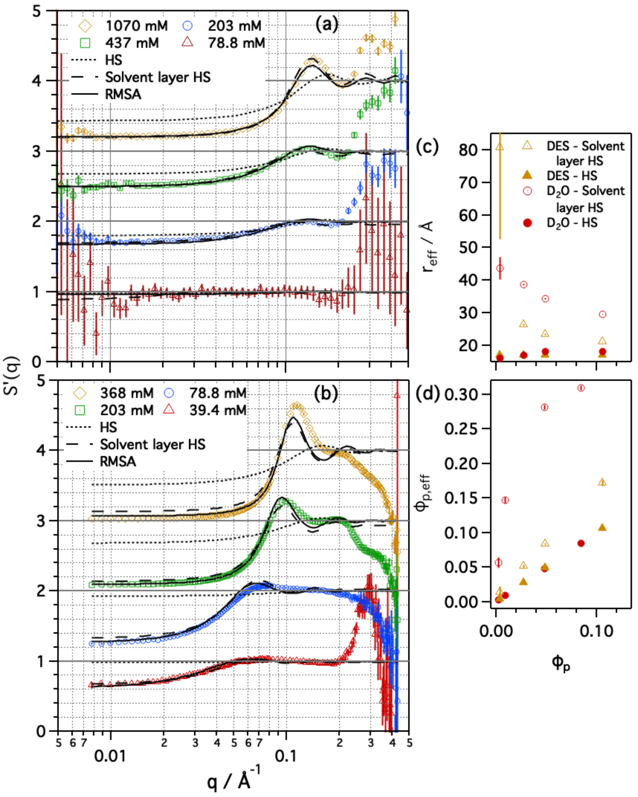

Figure S3 S(q)' data and best fits from different concentrations of h-C_{12}TAC micelles in (a) 1:2 d-ChCl:d-Glyc and (b) D_2O. The concentrations of surfactant are presented in the legend of graph (a) and (b). Data were modelled using the HS model (dotted lines), HS model including a solvent layer around the particle (dashed lines) and RMSA model (solid lines). Data and models were scaled for clarity. Variation of (c) r_{eff} and $\varphi_{p,eff}$ as a function of micelle volume fraction in DES and D_2O as shown in the legend of graph (c). Where not seen, the error bars are within the markers.

Table S4 Results from the analysis of the data of different concentrations of d-C_{12}TAB in h-ChBr:h-Glyc using a HS model that includes a solvent layer around the particle. The parameters derived from the analysis of the scattering data using the RMSA approach are included for comparison.

[C12TAC] / mM	r_{eff} / Å	$\phi_{p,eff}$ / ×10^{-2}	$r_{eff,P(q)}$ / Å	$\phi_{p,P(q)}$ / ×10^{-2}	Δr_{eff} / %	$\Delta\phi_p$ / %
1:2 d-ChCl:d-Glyc						
78.8	80.7±28.1	1.36±0.84	17.1±0.3	0.35±0.03	372±130	280±190
203	26.5±0.2	5.16±0.17	17.1±0.3	2.79±0.04	49±3	84±3
437	23.4±0.2	8.35±0.09	17.1±0.3	4.87±0.05	37±3	71±2
1070	21.1±0.1	17.2±0.4	17.1±0.2	10.6±0.1	23±1	61±2
D_2O						
39.4	43.8±3.5	5.63±0.71	16.1±0.4	0.23±0.03	173±14	2300±100
78.8	38.6±0.3	14.7±0.4	17.1±0.3	0.93±0.03	155±5	1500±100
203	34.4±0.2	28.1±0.3	18.1±0.3	4.82±0.02	90±3	480±10
368	29.6±0.1	30.9±0.3	18.3±0.2	8.49±0.02	61±1	260±10

Monomer concentration of counterion exchanged surfactants in DES

As the fitted micelle volume fractions in DES are lower than the total surfactant volume fraction, it is expected that a certain amount of solubilized surfactant monomer remains in solution. Using the total surfactant volume fraction (ϕ_s), as calculated from the surfactant concentration in the sample, and the fitted micelle volume fraction (ϕ_p), the volume fraction and concentration of surfactant in the monomeric form (ϕ_m and $[C_{12}TA^+]_m$, respectively) were calculated for each surfactant concentration. The density of the counterion-exchanged surfactants was approximated as 1 g/cm^3 for the calculations. The results from the calculations are presented in Table S5.

Table S5 Calculated monomer volume fraction and concentration of monomers for different surfactant concentrations of h-$C_{12}TA^{+}$ in 1:2 d-ChCl:d-Glyc: SO_4^{-2}, Br^{-}, NO_3^{-}, and Cl^{-}.

$[C_{12}TA^{+}]$ / mM	ϕ_s / $\times10^{-2}$	ϕ_p / $\times10^{-2}$	ϕ_m / $\times10^{-2}$	$[C_{12}TA^{+}]_m$ / mM
h-C_{12}TAC in d-ChCl:d-Glyc				
78.8	2.04	0.35±0.03	1.69±0.03	57.3±1.0
203	5.09	2.79±0.04	2.30±0.04	78.3±1.4
437	10.3	4.87±0.05	5.47±0.05	186±2
1070	22.0	10.6±0.1	11.4±0.1	387±4
h-C_{12}TAB in d-ChCl:d-Glyc				
79.7	2.43	1.08±0.03	1.34±0.03	45.7±1.0
205	5.60	3.60±0.05	2.00±0.05	68.2±1.7
422	11.4	7.01±0.08	4.39±0.08	149±3
943	23.4	12.6±0.1	10.8±0.1	367±3
h-$C_{12}TANO_3$ in d-ChCl:d-Glyc				
83.9	2.38	0.90±0.05	1.48±0.05	50.3±0.7
200	5.50	2.87±0.04	2.63±0.04	89.6±1.4
426	11.0	5.37±0.05	5.64±0.05	192±2
932	21.3	8.22±0.06	13.1±0.1	445±3
h-$C_{12}TA(SO_4)_{1/2}$ in d-ChCl:d-Glyc				
86.0	2.32	0.29±0.07	2.03±0.07	69.1±1.4
205	5.36	1.13±0.08	4.23±0.08	144±3
436	10.8	2.97±0.04	8.07±0.04	275±2
965	21.1	9.03±0.08	12.1±0.1	411±3

Modelling approach for determining Coulomb coupling constants

In order to directly compare the coupling constant for a given surfactant concentration, the experimental results for the coupling constant were modelled using an empirical approach for each of the studied surfactants. The Coulomb coupling constant versus the micelle volume fraction were fitted to the following equation:

$$\log(\Gamma_k) = a + b\log(\phi_p) \qquad (S1)$$

Subsequently, the Coulomb coupling constant of each system was evaluated for an arbitrary micelle volume fraction (ϕ_p). The results from the modelling are included in Figure S3. The models and calculations are presented in Table S4.

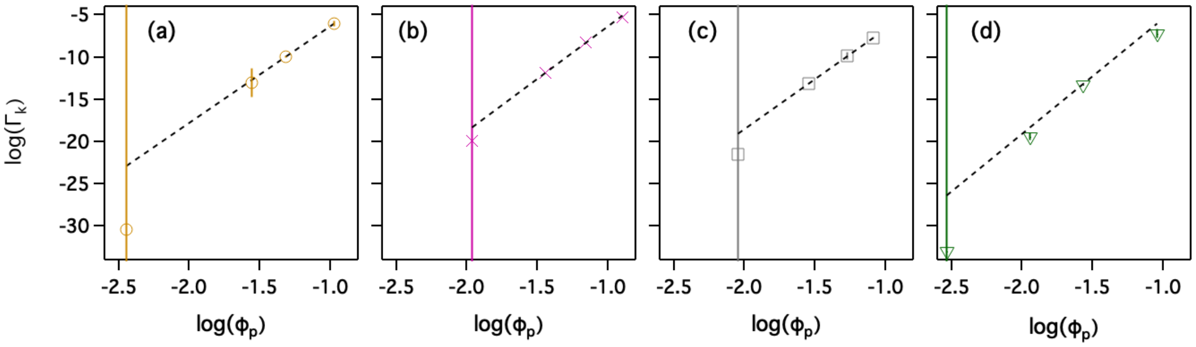

Figure S4 Modelling of the experimental Coulomb coupling constants for (a) h-C_{12}TAC, (b) h-C_{12}TAB, (c) h-$C_{12}TANO_3$, and (d) h-$C_{12}TA(SO_4)_{1/2}$ micelles in 1:2 d-ChCl:d-Glyc as a function of surfactant volume fraction. The models are presented as black dashed lines. Where not seen, the error bars are within the markers.

Table S6 Results from the modelling presented in Figure S3 using Equation S1. The Coulomb coupling constant was calculated for a volume fraction of micelles of 0.1 for comparison.

System	a	b	$\Gamma_k(\phi_p=0.1)$
h-C_{12}TAC in d-ChCl:d-Glyc	5.598±0.129	11.913±0.135	$4.841\times10^{-7}\pm9.626\times10^{-9}$
h-C_{12}TAB in d-ChCl:d-Glyc	5.430±0.109	11.905±0.091	$3.348\times10^{-7}\pm6.714\times10^{-9}$
h-$C_{12}TANO_3$ in d-ChCl:d-Glyc	5.183±0.086	11.856±0.117	$2.123\times10^{-7}\pm3.185\times10^{-9}$
h-$C_{12}TA(SO_4)_{1/2}$ in d-ChCl:d-Glyc	7.123±0.420	13.385±0.324	$5.467\times10^{-7}\pm1.219\times10^{-8}$

Variation of the Coulomb coupling constant with the solvent ionic strength

As seen in the main text, the strength of long-range interactions in DES is significantly reduced compared to those in water. This difference was attributed to the higher ionic strength of the solvent. Using Equation 5, theoretical calculations of the coupling constant were performed for a model particle with r_{eff}=18.35 Å, AR=1.3, z_p=40 and ϕ_p=0.085 in D_2O (ε=77.9, 30 ˚C). The coupling constant was determined for different solvent ionic strengths: 10, 25, 50, 100, 250 and 500 mM. Using the values of the Coulomb coupling constant, the theoretical scattering for the particles was determined using a uniform ellipsoid form factor and the RMSA model. The results from the calculations are presented in Table S7 and Figure S5. The theoretical scattering for the same particles interacting through excluded volume effects (HS) is presented for comparison.

Table S7. Theoretical predictions of the Coulomb coupling constant at different ionics strengths. The calculations were performed for a model particle with r_{eff}=18.35 Å, AR=1.3, z_p=40 and ϕ_p=0.085 in D_2O (ε=77.9, 30 ˚C). The value for hard sphere interactions is included for comparison.

I / mM	Γ_k
10	5.27E+02
25	5.90E+01
50	5.40E+00
100	2.01E-01
250	3.57E-04
500	3.40E-07
HS	0

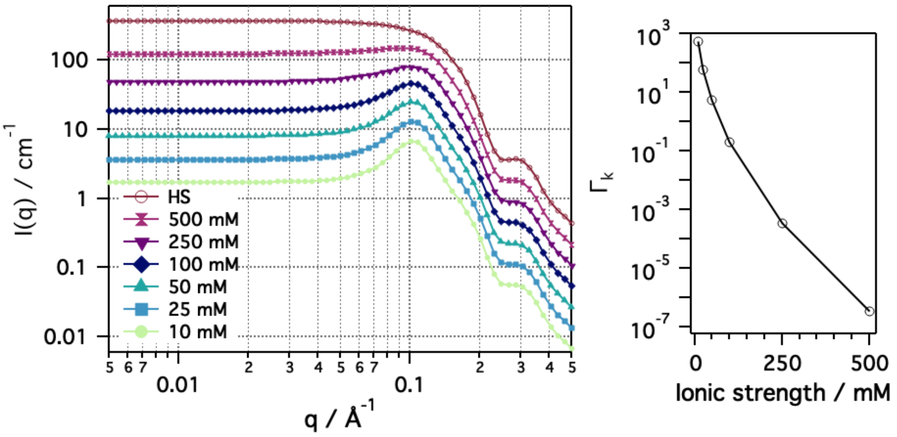

Figure S5. Calculated scattering intensities for the model particle interacting electrostatically through different the Coulomb coupling constants (see Table S5).

Sensitivity analysis of the correlation between the solvent ionic strength and particle charge

As introduced in the main text, the coupling between the solvent ionic strength and particle surface charge in the calculation of the Coulomb coupling constant limits the accuracy of the determination of these individual variables. To study this correlation, a sensitivity analysis was performed. Using the experimental data from 1070 mM C_{12}TAC in 1:2 d-ChCl:d-Glyc, the pairs of particle charge and solvent ionic strength were determined for the Coulomb coupling constant that fits the data. Initially, the aggregation number (N_{agg}) of the micelles was calculated using the equation:

$$N_{agg} = \frac{V_p}{V_m}$$

V_p is the volume of the micelle and V_m is the volume of the surfactant monomer. The V_m was calculated using Tanford equations and is 350.2 Å^3 (Tanford, *J. Phys. Chem.* 1972). The particle charge values were obtained considering a realistic upper and lower boundary for the counterion dissociation, i.e. α_{min}=20% and α_{max}=80%, where z_p=aN_{agg}. The model was parametrized using and ε=22.8, and r_{eff}=17.1 Å and N_{agg}=52±3 from the structural analysis of the micelles. Using these values in the analysis, the experimental data were fitted to obtain the Coulomb coupling constant, particle surface potential, and the effective ionic strength of the solvent. The results from the sensitivity analysis are presented in Table S8.

Table S8 Results from the sensitivity analysis of the correlation between the particle charge and solvent ionic strength.

α	z_p	ψ_0 / $\times 10^{-2}$ V	I / mM	Γ_k / $\times 10^{-7}$	χ^2
0.2	10.4	3.79±0.03	338±6	8.86±0.05	28.123
0.3	15.6	5.67±0.04	398±5	8.80±0.05	24.036
0.4	20.8	7.18±0.05	435±6	8.88±0.06	21.394
0.5	26.0	8.64±0.05	465±7	8.87±0.06	19.074
0.6	31.2	10.1±0.1	491±5	8.86±0.04	18.256
0.7	36.4	11.5±0.1	513±6	8.84±0.05	19.514
0.8	41.6	13.1±0.1	538±6	8.82±0.06	20.797

The analysis shows that the correlation between the surface potential and ionic strength results in pairs of values that give fits of relatively equal quality (χ^2 between 20 and 30). These pairs of values result in the same Coulomb coupling constant, as the contact potential for the interparticle repulsion is determined from the experimental scattering. The difference in the quality of the fits is marginal due to the correlation between the two parameters. However, it is seen that the effective ionic strength of the solvent is around 400-500 mM.

References

1. Sanchez-Fernandez, A.; Hammond, O. S.; Edler, K. J.; Arnold, T.; Doutch, J.; Dalgliesh, R. M.; Li, P.; Ma, K.; Jackson, A. J., Counterion binding alters surfactant self-assembly in deep eutectic solvents. *Phys. Chem. Chem. Phys.* **2018,** *20* (20), 13952-13961.
